# Speciation of Selenium in Brown Rice Fertilized with Selenite and Effects of Selenium Fertilization on Rice Proteins

**DOI:** 10.3390/ijms19113494

**Published:** 2018-11-06

**Authors:** Zhenying Hu, Yixin Cheng, Noriyuki Suzuki, Xiaoping Guo, Hua Xiong, Yasumitsu Ogra

**Affiliations:** 1 State Key Laboratory of Food Science and Technology, NanChang University, Nanchang 330047, China; whozing@gmail.com; 2 Laboratory of Toxicology and Environmental Health, Graduate School of Pharmaceutical Sciences, Chiba University, Chuo, Chiba 260-8675, Japan; n-suzuki@chiba-u.jp; 3 Jiangxi Institute for Drug Control, Jiangxi Province Engineering Research Center of Drug and Medical Device Quality, Nanchang 330029, China; cyx900306@163.com; 4 Jiangxi Research Center for Auxiliary Food Engineering and Technology, Ganzhou 341100, China; eeeefnhy@163.com

**Keywords:** selenium, brown rice, speciation, glutelin, foliar spray, ICP-MS

## Abstract

Foliar Selenium (Se) fertilizer has been widely used to accumulate Se in rice to a level that meets the adequate intake level. The Se content in brown rice (*Oryza sativa* L.) was increased in a dose-dependent manner by the foliar application of sodium selenite as a fertilizer at concentrations of 25, 50, 75, and 100 g Se/ha. Selenite was mainly transformed to organic Se, that is, selenomethionine in rice. Beyond the metabolic capacity of Se in rice, inorganic Se also appeared. In addition, four extractable protein fractions in brown rice were analyzed for Se concentration. The Se concentrations in the glutelin and albumin fractions saturated with increasing Se concentration in the fertilizer compared with those in the globulin and prolamin fractions. The structural analyses by fluorescence spectroscopy, Fourier transform infrared spectrometry, and differential scanning calorimetry suggest that the secondary structure and thermostability of glutelin were altered by the Se treatments. These alterations could be due to the replacements of cysteine and methionine to selenocysteine and selenomethionine, respectively. These findings indicate that foliar fertilization of Se was effective in not only transforming inorganic Se to low-molecular-weight selenometabolites such as selenoamino acids, but also incorporating Se into general rice proteins, such as albumin, globulin glutelin, and prolamin, as selenocysteine and selenomethionine in place of cysteine and methionine, respectively.

## 1. Introduction

Selenium (Se) is an essential trace element with various important biological functions in both humans and animals. However, the available resource of Se is limited in the world, and its distribution is quite uneven. The Se content in soil is closely related to ethnic health problems due to Se deficiency such as Keshan disease in China [1]. In this area, Se supplementation is one of the most effective measures against Se deficiency. The biological activity, nutritional availability, and toxicity of Se depend on its concentration and chemical species [2]. Although inorganic species of Se such as sodium selenite are more toxic than organic Se at the same concentration, they are widely used as a food additive and fertilizer for vegetables and crops because of their economical advantages [3]. Rice is one of the main crops in the world, particularly in Asia; thus, it could be a significant and potential resource of dietary Se in Se-deficient areas. As reported previously, Se could be enriched in rice during the period of paddy planting by foliar fertilization and irrigation with Se fertilizer [4]. Unexpectedly however, not all of the exogenous inorganic Se was transformed to organic Se, and nontransformed Se was stored in rice. Although the total Se contents in both rice shoots and roots of rice were increased by fertilizing with high concentrations of inorganic Se, the percentages of organic Se in the shoots and roots were decreased by the increase in the concentration of the applied Se fertilizer [5,6,7,8].

Selenate and selenite are widely used to increase the Se content in rice by foliar spraying [9]. It has been proved that selenate shows more potential accumulation for plants including rice than selenite. Selenate is the major form of Se in oxidized soil including most cultivated soil, whereas selenite predominantly exists in anaerobic soil with neutral to acidic pH including paddy soils [10]. Selenite is rapidly converted to organoselenium compounds after the absorption via phosphate transporters, and selenate is delivered immediately to the xylem after the absorption via sulphate transporters, and then, reduced to selenite by adenosine 5’-phosphosulphate and assimilated into organoselenium compounds in plastids [11,12,13]. The conversion of selenate to selenite seems to be the rate-limiting step in the assimilation of Se into organic compounds [14]. Hence, we used selenite rather than selenate in this experiment. After its absorption by plants, selenite is reduced to selenide, which is then transformed to selenocysteine (SeCys) and selenomethionine (SeMet), and a portion of selenoamino acid is incorporated into protein [15,16]. Se is not an essential element for plants including rice [17], but it could be taken up by plants owing to its physicochemical similarity to sulfur (S) and affects the S metabolism in plants [15]. The mistranslation of SeCys and SeMet into plant proteins instead of cysteine (Cys) and methionine (Met), respectively, is thought to be the cause of phytotoxicity of Se in plants because it is expected to disrupt protein folding resulting in the dysfunction of plant proteins [18]. Rice proteins can be divided into four fractions, namely, albumin, globulin, glutelin, and prolamin according to their solubility in different solvents [19]. Because globulin and prolamin contain larger amounts of S than glutelin [20], they seem to have a higher potential to accumulate Se via S metabolism. However, this concept remains controversial. For example, Aureli et al. found that almost 80% of total Se is incorporated in the water insoluble protein fraction of rice [21]. Fang et al. revealed that the glutelin faction, which is the main storage protein in rice, contained the largest amount of Se, approximately 31.3% of total Se, and followed by the albumin (9.7%), globulin (7.0%), and prolamin (6.0%) fractions in this order [22]. On the other hand, Zhang et al. suggested that the Se content in protein fractions decreases in the order of glutelin, prolamin, albumin, and globulin [23]. Taken together, it should be clarified how Se is metabolized and accumulated in rice, and whether the mistranslation has any effects on rice proteins.

In this work, we analyzed the Se content and species in brown rice, Zhuliangyou 819 (*Oryza sativa* L.), with foliar fertilization of sodium selenite at several concentrations and evaluated the effects on the structure of Se-containing glutelin. We intended to demonstrate the Se metabolism in rice by speciation analysis using high performance liquid chromatography with inductively coupled plasma mass spectrometry (LC-ICP-MS). Moreover, we examined the structural changes of Se-containing glutelin by fluorescence spectrometry, Fourier transform infrared spectrometry (FT-IR) and differential scanning calorimetry (DSC).

## 2. Results

### 2.1. Se Concentration in Brown Rice

**Figure 1:**
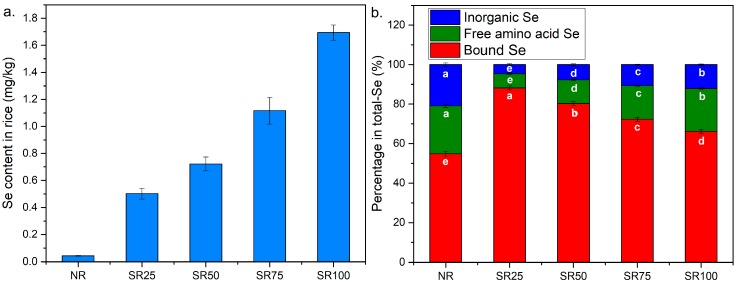
Se content in rice (**a**) and the percentages of concentrations of inorganic Se, free amino acid Se, and bound Se to that of total selenium in rice water extract (**b**). Within each column, values followed by different letters are significantly different from one another (*p* < 0.05).

The final concentrations of treatment with sodium selenite were set at 0, 25, 50, 75, and 100 g Se/ha, and the rice classified into the NR, SR25, SR50, SR75, and SR100 groups, respectively. The foliar Se fertilizer increased the Se concentration in brown rice in a dose-dependent manner (Figure 1a). In the control rice (NR), the Se concentration was 0.043 mg Se/kg. The Se concentrations of SR25, SR50, SR75, and SR100 were 0.502, 0.722, 1.117, and 1.694 mg Se/kg, respectively. Figure 1b shows the percentages of concentration of inorganic Se, free amino acid Se, and bound Se with respect to the total concentration of Se rice obtained by water extraction. Bound Se was composed of organic Se bound to protein, starch, and lipids, which were difficult to extract with water. The contents of organic Se, inorganic Se, and protein-bound Se increased after treatment, but the percentage of organic Se concentration with respect to total Se concentration increased in the range from 0 to 25 g Se/ha and decreased in the range from 25 to 100 g Se/ha. Approximately 79.6% of Se absorbed by plants was stored in the organic form, that is, Se in free amino acids and unextracted, without Se treatments in our study. With the increase in Se concentration in the foliar fertilizer, the content of organic Se was increased by biofortification. The ratio of inorganic Se to total Se returned to 12.01% at the concentration of 100 g Se/ha, but still lower than that of the control (20.88%). It was revealed that at the concentration of 25 g Se/ha, fortified Se was efficiently transformed to organic Se in rice. However, the efficiency of conversion of organic Se is not increased with concentration of Se in the foliar fertilizer.

### 2.2. Se Species in the Water Extract Identified by LC-ICP-MS

**Figure 2:**
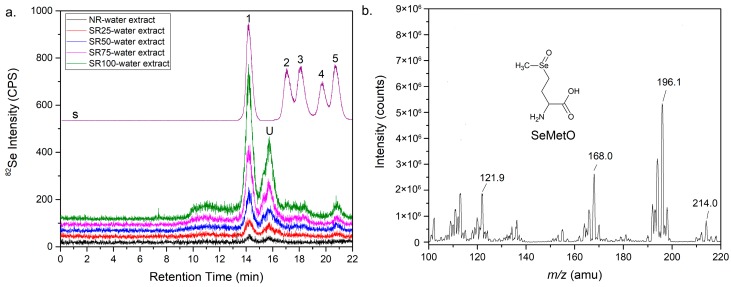
Profiles of standard blend (violet line. s) of selenate (1), selenite (2), SeCys2 (3), MeSeCys (4), and SeMet (5) at 100 ng Se mL^−1^ and water extract of rice obtained by HPLC-ICP-MS (**a**). Identification of unknown peak corresponding to component eluted from water extract by ESI mass spectroscopy (**b**).

LC-ICP-MS provided sufficient separation thereby enabling us to identify the possible Se species in rice (Figure 2a). The retention times of standard selenate, selenite, SeCys2, *Se*-methylselenocysteine (MeSeCys), and SeMet were 14.5, 17.0, 18.0, 19.6, and 20.6 min, respectively (Figure 2a). The largest peak among those at all Se concentrations was observed at a retention time of 14.5 min corresponding to selenate. The second largest peak corresponded to a component eluted at a retention time of 15.7 min, which did not match with any of those of standards, suggesting that the second largest peak corresponded to Se compounds different from the standards we used. It is difficult to identify Se species by LC-ICP-MS when standard compounds are not available. Thus, the component in the water extract corresponding to the unknown peak was analyzed by electrospray ionization mass spectrometry (ESI-MS) under positive mode. We obtained Se isotopic signals at *m/z* 121.9, 168.0, 196.1, and 214.0 as ^80^Se (Figure 2b). These fragments were assignable to selenomethionine selenoxide (SeMetO), as shown in Table 1, which is an oxidation product of SeMet [24]. Minor peaks corresponding to selenite, SeCys2, and SeMet were detected, but no apparent peak corresponding to MeSeCys was detected even at the highest Se concentration. These results indicate that the main Se species existing as low-molecular-weight Se metabolites in the water extract were selenate and SeMetO. Although selenite and SeMet were detected as minor peaks, major portions of these metabolites were oxidized to selenate and SeMetO, respectively, during their preservation, preparation, and extraction. Selenite is an intact form in the fertilizer; thus, SeMet seems to be the main metabolite in Se-fortified rice. 

### 2.3. Distribution of Se in Extracted Fractions

**Figure 3:**
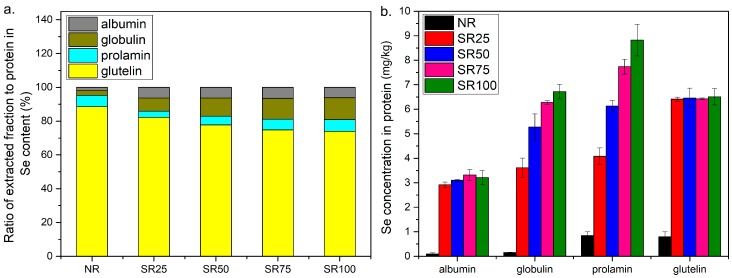
Ratio of Se in different fractions to total protein-Se (**a**) and concentration of Se in different fractions (**b**).

Se exists mainly in the glutelin fraction, accounting for 88.62% in protein in rice without selenite treatment (NR). The percentage of Se in glutelin decreased to 73.90% when foliar fertilization by spraying at 100 g of Se/ha was conducted. On the other hand, the percentage of Se in the globulin fraction increased from 2.9% to 12.89% with increasing concentration of selenite for treatment (Figure 3a). The Se concentrations in four kinds of protein fractions in rice are shown in Figure 3b. The Se concentration in the control increased in the protein fraction in the following order: albumin (0.38 mg/kg) < globulin (0.47 mg/kg) < prolamin (0.80 mg/kg) and glutelin (0.79 mg/kg). After biofortification, the Se concentration in the protein fraction increased with the Se concentration in the fertilizer. The Se concentration in the albumin and glutelin fractions did not change and likely saturated to the Se concentration in the selenite treatment. The Se concentration in the globulin and prolamin fractions increased with the Se concentration in the fertilizer. The Se concentration in prolamin fraction was higher than that in the glutelin fraction when 75 and 100 g Se/ha was applied. 

### 2.4. Se Species in the Glutelin and Prolamin Fractions

SeCys2 was the main Se species in the prolamin and glutelin fractions in NR (Table 2). After the Se treatment, selenite was efficiently transformed to SeMet in the glutelin fraction. The ratio of SeMet to total selenoamino acids (SeMet plus SeCys2) in the glutelin fraction was in the range from 82.8% to 94.8% in rice with Se treatment. In contrast to the glutelin fraction, the ratio of SeCys2 to total selenoamino acids was relatively high in the prolamin fraction. Unlike in the glutelin fraction, the concentration of SeMet in the prolamin fraction was in a Se-concentration-dependent manner. The concentration of SeCys2 was saturated at the higher doses (around 50 g Se/ha treatment).

### 2.5. Amino Acid Compositions in the Glutelin Fraction

The amino acid compositions in the glutelin fraction are listed in Table 3. Glutamic acid is the most abundant amino acid in the fraction, followed by arginine, leucine, and alanine. The contents of S-containing amino acids, namely, Cys and Met, in the fraction were affected by the Se treatment. The Met content significantly decreased depending on the Se concentration in the fertilizer. In contrast to Met, the Cys content increased from 3.50 ± 0.22 to 4.58 ± 0.17 g/100 g protein in the range from 0 to 75 g Se/ha despite the treatment at the concentration of 100 g Se/ha, which was significantly higher than that of the control (NR, 3.50 ± 0.22). Although almost all amino acids except proline in the glutelin fraction were affected by the Se treatment, the amounts of essential amino acids did not apparently change. Threonine, one of the limited amino acids, was significantly enriched at the higher concentration of Se (75 and 100 g Se/ha). 

### 2.6. Fluorescence Spectra of the Glutelin Fraction

**Figure 4:**
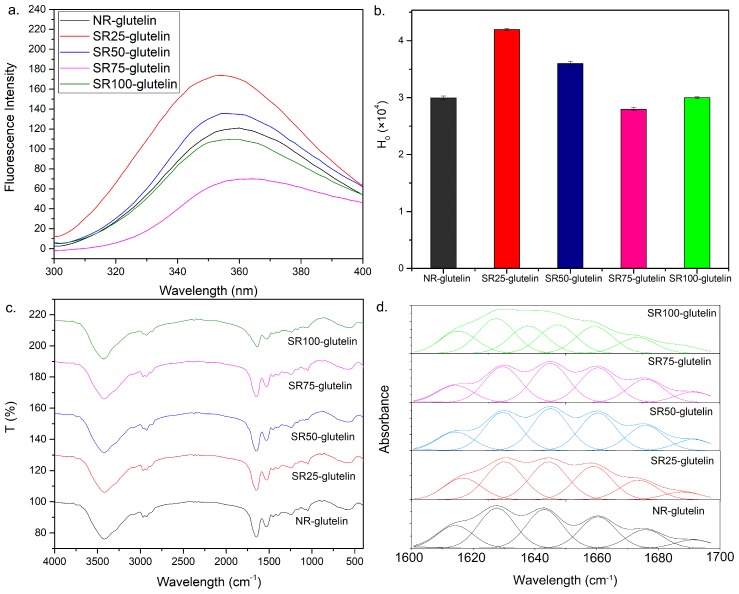
Intrinsic fluorescence (**a**), H_0_ of Se-containing glutelin fraction from different plant groups (**b**), FT-IR analysis of Se-containing glutelin fraction from different plant groups (**c**), and deconvoluted FT-IR spectra and curve fitting of glutelin fraction in the wavelength range of 1600–1700 cm^−1^ (**d**).

Aromatic amino acids in a protein such as phenylalanine and tyrosine have intrinsic fluorescence. Since the *λ*_max_ of fluorescence emission is affected by their structure and condition, the shift of *λ*_max_ could provide information and show changes of protein tertiary conformation. With the red and blue shifts of *λ*_max_ of emission compared with those of the Se-containing glutelin fraction in NR, the intrinsic fluorescence spectra revealed that the conformation of the glutelin fraction was affected after Se treatment (Figure 4a). However, there were no significant differences (*p* > 0.05) in the surface hydrophobicity (H_0_) of glutelin among NR, SR75, and SR100. The H_0_ of glutelin in SR25 and SR50 were higher than those in other plant groups (Figure 4b). Changes in H_0_ may be due to the differences in the structures of ANS-protein complexes. It was revealed that more hydrophobic structures were exposed in Se-containing glutelin.

### 2.7. Secondary Structures of the Se-Containing Glutelin Fractions

Absorbance between 1480 and 1200 cm^−1^ in FT-IR spectra are considered the fingerprint area of protein. There was no characteristic absorption observed in samples, as determined from FT-IR spectra between 4000–400 cm^−1^ (Figure 4c). This indicates that no new bonds were created, and/or the absorbance overlapped with other peaks. The FT-IR spectra in the 1700–1600 cm^−1^ region of the protein was deconvoluted to determinate the content of secondary structures by analyzing the amide I band known as the stretching vibration of C=O, which pertains to in-plane NH bending and CN stretching. The fitting results are shown in Figure 4d and the contents of secondary structures are shown in Table 4. Six peaks were fitted in the range of 1600–1700 cm^−1^ in all samples. However, the band shifted distinctly when compared with those in NR, especially in SR100. This observation suggests that the glutelin structure changed after Se treatment. The α-helix in glutelin was higher than in the control after Se treatment. Many β-structures and random coils turned in to α-helix after treatment at a high Se concentration. These findings suggest that the Se treatment affected the structure of glutelin and enriched the α-helix structure.

### 2.8. Contents of Sulfhydryl Groups (SH) and Disulfide Bond (S-S)

**Figure 5:**
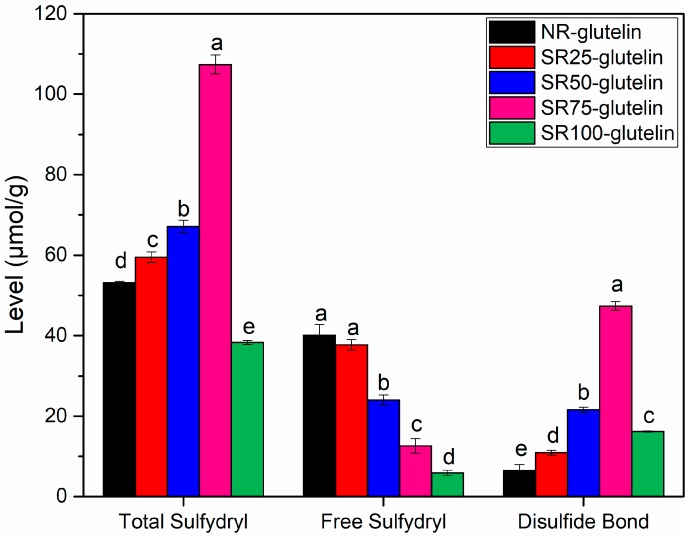
Contents of total sulfhydryl (SH_T_), free sulfhydryl (SH_F_), and disulfide bond (S-S) in glutelin fraction from different plant groups. Within each column, values followed by different letters are different (*p* < 0.05) from one another.

Free thiols and selenol species were quantified by monitoring the increase in absorbance at *λ*_max_ of 412 nm, which is due to the liberation of 2-nitro-5-thiobenzoate (TNB) upon the reactions between nucleophilic thiol and selenol species [25]. Note that the Se content in the glutelin fraction was extremely lower than the S content; thus, the interference of selenol species could be negligible. As shown in Figure 5, the content of the free sulfhydryl group in the glutelin fraction decreased with the increase in the concentration of Se for treatment, which corresponded to the amount of S-containing amino acids. This might indicate the replacement of S to Se because these elements share the same metabolic pathway [22]. 

### 2.9. DSC Analysis

The results of the DSC analysis of glutelin thermostability are summarized in Table 4. After Se treatment, the initial temperatures (*T*_o_), final temperatures (*T*_e_) and temperature peak maximum (*T*_p_) of endothermic transition showed slight difference. In addition, the enthalpy of denaturation to glutelin increased with the concentration of selenite for treatment in the range from 0 to 75 g Se/ha. It seemed that the thermostability of glutelin increased after Se treatment to some extent. However, *∆H* of glutelin in the SR100 group decreased to 18.62 J/g. This might be the cytotoxic effect on rice and the structural disorder of protein at a very high concentration of Se.

The total amount of sulfhydryl was increased by the Se treatment (Figure 5). Taking the results together, the Se treatment could enhance the thermostability of glutelin at a certain Se concentration (Table 4), and improve protein denaturation when the applied Se concentration was too high.

## 3. Discussion

The cultivar and spraying method used, the planting environment, and the Se species in a fertilizer contribute to the enrichment of Se in plants [26]. Foliar spraying is more effective than soil application in terms of biotransformation from inorganic Se to organic Se, selenite foliar spraying during the heading stage can increase the Se content in agricultural crops such as rice and wheat [26,27]. In the non-Se-biofortified rice plants, which served as the control, selenate and SeMet were the main freeform Se species (≥44.5%). It was also shown that Se incorporated from soil to plant roots remained as inorganic Se in nonsupplemented rice [28]. Se is a nonessential element for plants; thus, it seems that no metabolism of Se occurs below the toxic concentration of inorganic Se in crops. On the other hand, this study showed that inorganic Se was not sufficiently transformed to organic Se and incorporated into proteins at high Se concentrations, suggesting that Se in the fertilizer, that is, selenite, was metabolized into organic forms such as SeCys and SeMet within the metabolic capacity. Indeed, the ratios of SeCys and SeMet in the forms of free amino acids and bound to proteins were increased by the Se treatment at the lower concentration (Figure 1b and Figure 3b). Since SeCys and SeMet were susceptible to oxidation, they were transformed into SeCys2 and SeMetO, respectively, during the preparation and extraction. Wang et al. [5] suggested that the percentage of organic Se compounds in shoots and roots decreased when more than 2 mg/L sodium selenite was applied. Our observation (Figure 1b) was in agreement with the literature. Organic and inorganic Se accumulated in the roots are transported to the grains via the phloem [29]. Therefore, there is the preferable accumulation of inorganic Se and free selenoamino acids in rice grains.

**Figure 6:**
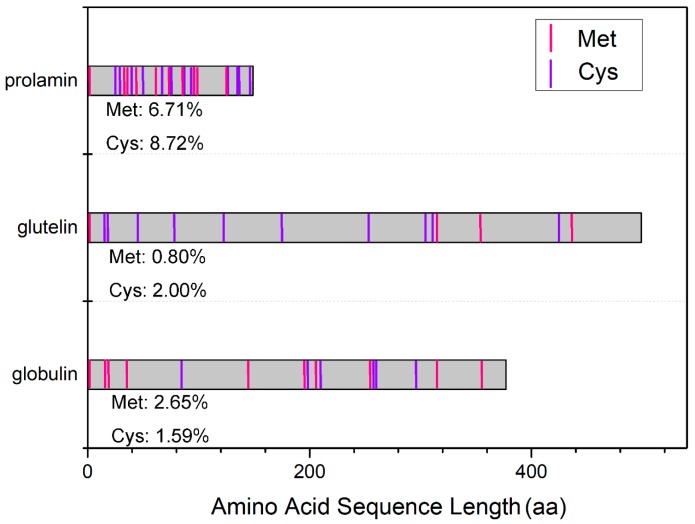
Amino acid sequence lengths and related locations of Met (red mark) and Cys (violet mark) in proteins based on NCBI database.

Prolamin and glutelin are the storage proteins in rice [30]. Glutelin accounts for 70–80% of total rice endosperm protein, and prolamin is only 2.46% of total rice protein. They contain large amounts of Se in rice [21,22]. Thus, the main Se storage in rice is glutelin. Indeed, Se was highly accumulated in glutelin (Figure 4b). It was speculated that Se is taken up by plants via the metabolic pathway shared with S, because both elements have physicochemical similarities. Then, Se is also incorporated into general proteins as SeCys and SeMet instead of Cys and Met [15]. Moreover, the different efficiencies of SeMet transport from flag leaves to grains resulting in various degrees of Se enrichment in the grains were reported [31]. Gong et al. [32] indicated that the transport efficiency of selenoamino acids in rice probably contributes to the differences in the ratio of SeMet to Met in glutelin. However, the Se concentration in glutelin did not increase with the increase in the Se concentration in the fertilizer (Figure 3b). Glutelin showed the lowest S content among the four protein fractions (Figure 6), which is in agreement with a previous observation [33]. Thus, glutelin could be more easily saturated with Se than the other fractions. Although the Se concentration in glutelin was indeed lower than that in prolamin, the Se content in glutelin mainly contributed to the total Se amount in rice because the content of glutelin is the highest (Figure 3b). In contrast to glutelin, the Se concentrations in prolamin and globulin linearly increased up to a higher Se concentration in the fertilizer since these two fractions contained more thioamino acids (Figure 6). These indicate that prolamin and globulin also contribute to the tolerance to Se toxicity in rice owing to their high capacities for selenoamino acids. Dhanjal et al. [34] found that prolamin and globulin contained larger amounts of Se than glutelin and albumin, and prolamin showed a high capacity for Se at the high Se concentration treatments in wheat, maize, and rice. 

With the application of Se, more SeMet was synthesized in the glutelin fraction and the amount of SeCys2 only slightly increased (Table 2). As mentioned above, SeMet replaced Met in glutelin fraction resulting in the decrease in Met amount [22]. Nevertheless, some reports showed that selenate treatment induced the accumulation of S in strawberry, wheat, and lettuce [35,36,37]. Paulo et al. revealed that the Se treatment caused S deficiency and induced the expression of the sulfate transporter resulting in S uptake [38]. Although selenite was used in this study as a fertilizer, the same phenomenon, that is, the increase in the S content by selenite treatment, was observed. In the present study, we applied selenite by the foliar spraying, suggesting that portions of selenite could be oxidized to selenate. Despite the decrease in the amount of Met in rice, that of Cys increased in the glutelin fraction (Table 3). Cys is the only amino acid that provides a sulfhydryl group and disulfide bonds. Hence, the increase in the Cys amount in the glutelin fraction affected the thermostability of glutelin (Figure 5 and Table 4). The changes in the amino acid composition, particularly the ratio of hydrophobic amino acids, could also affect the secondary and tertiary structures of protein (Table 3 and Figure 5).

In conclusion, the foliar Se fertilizer significantly increased the Se content in rice. However, not all of Se was transformed to an organic Se compound or incorporated into general proteins even at a high Se concentration in the fertilizer. The glutelin fraction was the main storage of Se in rice. Based on Se speciation, SeMet incorporated into proteins was the main Se form in rice after the Se treatment. Excess amounts of Se remained as inorganic Se and SeMet as a free amino acid (not incorporated into proteins); these Se compounds were detected as selenate and SeMetO owing to their oxidation. On the other hand, the content of Cys increased in the glutelin fraction because Se could affect the S metabolism. It could contribute to the enhancement of thermostability in the glutelin fraction.

## 4. Materials and Methods

**Chemical reagents.** Sodium selenite and SeMet were purchased from Nacalai Tesque (Kyoto, Japan). *Se*-Methylseleno-l-cysteine (MeSeCys) was purchased from Acros Organics (Waltham, MA, USA). l-Selenocystine (SeCys2) and sodium selenate were purchased from Tokyo Chemical Industry Co., Ltd. (Tokyo, Japan) and Wako Pure Chemical Industries, Ltd. (Osaka, Japan), respectively. SeMetO was synthesized based on the method of Kubachka et al. [39]. 8-Anilino-1-naphthalenesulfonic acid (ANS) and 5,5′-dithiobis (2-nitrobenzoic acid) (DTNB) were purchased from Sigma-Aldrich (Shanghai, China). All chemicals were of reagent grade or higher. Ultrapure deionized water (DIW) with 18.3 MΩ cm resistance was used and was prepared using a Milli-Q system (Merck Millipore, Tokyo, Japan). 

### 4.1. Plant Materials, Growth Conditions, and Pretreatment of Se-Containing Rice Samples

The field experiment described in detail by Fang et al. was carried out with some modification [40]. In brief, rice seeds (Zhuliangyou 819, *Oryza sativa* L.) purchased from a local agrotechnical station were planted in the field at Nanchang (N 28°26′, E 116°26′), Jiangxi Province in China on 9 April 2017 and hand harvested according to the plant groups on 18 July 2017. The pH of the soil region was 7.1, and the total Se content in the soil was 0.10 mg/kg of soil. The foliar fertilizer was composed of only sodium selenite and distilled water. Rice plants were treated at four Se concentrations (33.3, 66.7, 100, and 133.3 mg Se/L) once during the heading stage of growth. Control rice plants were sprayed with only distilled water on 8 June 2017. The final concentrations of treatment with sodium selenite were set at 0, 25, 50, 75, and 100 g Se/ha, and the plants classified into the NR, SR25, SR50, SR75, and SR100 groups, respectively. Each treatment was performed in three plots separated by a space of 0.5 m. Each plot had a size was 40 m^2^ (8.0 m × 5.0 m). Paddy transplantation, irrigation, and other rice farming practices were carried out on the basis of the farmers’ experience. After dehulling using a Satake rice machine (Type THU, Satake Engineering Co., Tokyo, Japan), brown rice grains were grounded and stored at −80 °C.

### 4.2. Extraction and Determination of Se Content in Se-Containing Brown Rice

**Figure 7:**
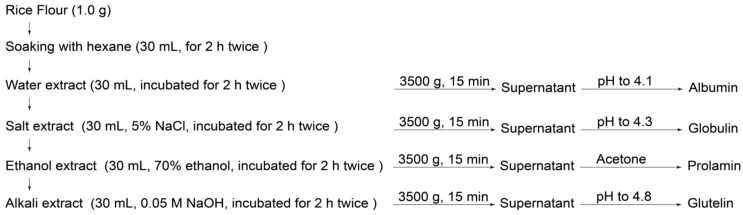
Illustration for separation procedure for protein fractions in rice, namely, albumin, globulin, prolamin, and glutelin, extracted with water, 5% NaCl, 70% ethanol, and 0.05 M NaOH, respectively.

The protein fractions in Se-containing brown rice were extracted according to the method of Fang et al. [22] with some modifications as shown in Figure 1. A 1 g portion of defatted brown rice powder was soaked in 30 mL of DIW under ultrasonic water bath (US-4R, BioRad, Tokyo, Japan) at 620 W for 30 min, and then centrifuged at 5000× *g* for 15 min. The supernatant after 0.45 μm syringe filtration was subjected to Se speciation analysis. Albumin, globulin, prolamin, and glutelin were extracted with DIW, 5% NaCl, 70% ethanol, and 50 mM NaOH, respectively, at a pH adjusted to the isoelectric point. The extraction procedure shown in Figure 7, was repeated twice. After freeze-drying, the protein fractions were stored at −20 °C until their analyses mentioned below. The purities of Se-containing proteins were determined more than 87.8% by the Kjeldahl method. The Se content in the Se-containing rice and protein fractions were determined by ICP-MS (7700cx, Agilent Technologies, Hachiouji, Tokyo, Japan).

### 4.3. Extraction of Selenocompounds and Se Speciation Analysis

Samples for extraction and Se speciation analysis were prepared by the method described previously with some modification [41]. A 0.2 g sample was precisely weighed into a plastic centrifuge tube, followed by the addition of 20 mg of protease K and 5 mL of DIW. The tubes were kept in the dark on a shaker for 24 h at 37 °C. The supernatants obtained by centrifugation of the hydrolyzed samples at 105,000× *g* for 20 min were passed through a 0.45 μm filter and stored at −20 °C until use.

A 100 μL aliquot of an extract was applied to a multimode gel filtration column (GS-320HQ; size exclusion, 40,000 Da; 7.5 i.d. × 300 mm; with a guard column, 7.5 i.d. × 75 mm, Showa Denko, Tokyo, Japan) and the column was eluted with 25 mM ammonium acetate, pH 6.5, at a flow rate of 0.6 mL/min. The eluate was introduced directly into a nebulizer tube of an ICP-MS (LC-ICP-MS) system and Se was monitored at *m/z* 82.

### 4.4. Identification of Unknown Se Compound

The eluted solution with the unknown Se compound, whose retention time was approximately 15.7 min as determined by GS-320HQ-HPLC mentioned in the Se speciation analysis section, was collected and concentrated by freeze-drying. The structure of the unknown Se compound was identified by the deconvolution of the fragment ions obtained by hybrid triple-quadrupole/linear ion trap mass spectrometry (4000 QTrap, AB Sciex, Foster City, CA, USA) under positive mode.

### 4.5. Amino Acid (AA) Analysis

AA analysis was performed according to the method by Lei et al. [42] with minor modification. A Se-containing glutelin sample (75 mg) was hydrolyzed with 10 mL of 6 M HCl and 1 g of phenol at 110 °C for 22 h. Then, samples were loaded into an automatic amino acid analyzer (L-8800, Hitachi, Tokyo, Japan) to determine the content of AA (g/ 100 g).

### 4.6. Fluorescence Measurements

Fluorescence spectra and surface hydrophobicity (H_0_) were determined as described by Wang et al. [43] with minor modification. Samples were dispersed in 50 mM PBS (pH 7.5) and centrifuged at 8000× *g* for 5 min. Then, the protein concentration of supernatants was adjusted to 0.01% (*m*/*v*). The fluorescence intensity of each sample was determined at excitation and emission wavelengths of 290 and 350 nm, respectively. H_0_ was determined using ANS as the fluorescence probe. In brief, 4 mL of supernatant with various protein concentrations from 0.001% to 0.01% (*w*/*v*) in 50 mM PBS (pH 7.0) was mixed with 10 µL of ANS (8 mM) for 1 h. The fluorescence intensities of supernatants with various protein concentrations were determined at excitation and emission wavelengths of 390 nm and 480 nm, respectively. The initial slope of fluorescence intensities versus protein concentrations (%, *w*/*v*) was used as H_0_.

### 4.7. Fourier Transformed Infrared (FT-IR) Spectroscopy 

FT-IR spectroscopy was carried out using a FT-IR spectrometer (Nicolet 5700, Thermo Nicolet Co., Madison, WI, USA) as described by Zhao et al. [44] Glutelin powder (2 mg) was mixed with KBr (200 mg) and pressed into pellets. Spectra were obtained in the wave number range from 4000 to 400 cm^−1^ during 32 scans at 4 cm^−1^. Data were analyzed using Peakfit 4.12 (Systat Software, San Jose Co., San Jose, CA, USA).

### 4.8. Contents of Total and Free Sulfhydryl (SH) Groups and Disulfide bonds

SH groups and the S-S group level of Se-containing glutelin were determined using Ellman’s reagent (DTNB) in accordance with the method of Zhao et al. [44] The contents of SH and S-S groups are presented as µmol/g protein.

### 4.9. Differential Scanning Calorimetry (DSC)

DSC measurements were performed using a Perkin Elmer Pyris Diamond DSC instrument (Perkin Elmer, Waltham, MA, USA). In brief, 6.5 mg of Se-containing glutelin samples with moisture of less than 6% was weighed on aluminum pans and sealed, then heated in a temperature ranged from 0–160 °C at a rate of 10 °C/min with an empty aluminum pan as the reference. The onset (*T*_o_), denaturation (*T*_p_), and end temperatures (*T*_e_), and denaturation enthalpy (*∆H*) were calculated using the instrument’s software.

### 4.10. Statistical Analysis

The results are presented as mean ± standard deviation (SD). One-way analysis of variance (ANOVA) was used to establish the significance of differences among groups and exposure period. A level of 0.05 was accepted as significant (*p* < 0.05). All statistical analyses were performed with Statistical program (SPSS 20.0, SPSS Inc., Chicago, IL, USA).

## Figures and Tables

**Table 1:** Parent ions and fragmentation products of SeMet and SeMetO signals at *m/z* as ^80^Se.

Compound	Parent *m/z*	Product *m/z*
SeMet	198	181 (-NH_3_) 153 (-COOH) 135 (-COOH, -NH_3_) 109 (CH_3_SeCH_2_^+^)
SeMetO	214	196 (-H_2_O) 168 (-CO) 150 (-H_2_O) 122 (-CH_2_CH_2_)

**Table 2:** Concentrations of Se species in glutelin and prolamin fractions.

Sample	Se Species in Glutelin (μg/g)		Se Species in Prolamin (μg/g)	
	SeMet	SeCys2	SeMet	SeCys2
NR	0.321 ± 0.007 ^c^	0.318 ± 0.006 ^b^	0.333 ± 0.040 ^e^	0.379 ± 0.012 ^c^
SR25	4.695 ± 0.041 ^b^	0.475 ± 0.006 ^a^	2.289 ± 0.008 ^d^	0.976 ± 0.026 ^b^
SR50	4.705 ± 0.022 ^b^	0.457 ± 0.021 ^a^	3.765 ± 0.244 ^c^	1.137 ± 0.077 ^a^
SR75	4.666 ± 0.024 ^b^	0.474 ± 0.089 ^a^	5.044 ± 0.029 ^b^	1.145 ± 0.022 ^a^
SR100	4.761 ± 0.028 ^a^	0.447 ± 0.075 ^a^	5.914 ± 0.122 ^a^	1.142 ± 0.066 ^a^

Data are expressed as the mean of three determinations ± standard deviation. In each column, values followed by different superscript letters are significantly different from each other (*p* < 0.05).

**Table 3:** Amino acid composition of glutelin fraction (g/100 g protein).

Amino Acid	NR-Glutelin	SR25-Glutelin	SR50-Glutelin	SR75-Glutelin	SR100-Glutelin
Asp	2.35 ± 0.01 ^b^	3.11 ± 0.59 ^a^	3.56 ± 0.27 ^a^	3.35 ± 0.38 ^a^	3.00 ± 0.18 ^a^
Thr	3.63 ± 0.15 ^b^	3.30 ± 0.11 ^b^	3.06 ± 0.16 ^b^	5.78 ± 0.85 ^a^	5.29 ± 0.45 ^a^
Ser	4.51 ± 2.08 ^bc^	7.31 ± 0.55 ^a^	5.63 ± 0.13 ^ab^	3.16 ± 0.11 ^c^	7.10 ± 0.50 ^a^
Glu	12.71 ± 3.23 ^b^	16.55 ± 0.30 ^a^	18.79 ± 0.10 ^a^	8.27 ± 1.29 ^c^	17.88 ± 0.22 ^a^
Gly	5.82 ± 0.30 ^a^	5.50 ± 0.08 ^b^	4.72 ± 0.02 ^c^	4.58 ± 0.17 ^c^	4.90 ± 0.14 ^c^
Ala	7.52 ± 0.78 ^a^	6.35 ± 0.12 ^b^	5.96 ± 0.03 ^b^	8.08 ± 0.64 ^a^	6.09 ± 0.22 ^b^
Cys	3.50 ± 0.22 ^bc^	3.73 ± 0.13 ^b^	3.39 ± 0.014 ^c^	4.58 ± 0.17 ^a^	2.40 ± 0.11 ^d^
Val	6.80 ± 0.40 ^a^	5.86 ± 0.08 ^b^	6.21 ± 0.03 ^b^	7.10 ± 0.30 ^a^	6.09 ± 0.22 ^b^
Met	2.73 ± 0.15 ^b^	2.93 ± 0.10 ^a^	1.99 ± 0.01 ^c^	1.79 ± 0.01 ^d^	1.95 ± 0.02 ^cd^
Ile	4.98 ± 0.32 ^a^	4.09 ± 0.12 ^b^	4.47 ± 0.02 ^b^	5.19 ± 0.20 ^a^	4.54 ± 0.24 ^b^
Leu	9.60 ± 0.57 ^a^	8.36 ± 0.07 ^b^	8.69 ± 0.04 ^b^	9.82 ± 0.43 ^a^	8.53 ± 0.32 ^b^
Tyr	5.96 ± 0.42 ^ab^	5.44 ± 0.18 ^c^	5.71 ± 0.03 ^b^	6.42 ± 0.23 ^a^	5.54 ± 0.20 ^d^
Phe	6.20 ± 0.37 ^b^	5.07 ± 0.07 ^c^	6.04 ± 0.17 ^b^	6.91 ± 0.31 ^a^	6.00 ± 0.16 ^b^
His	4.38 ± 0.30 ^b^	3.97 ± 0.04 ^c^	4.47 ± 0.02 ^b^	5.31 ± 0.13 ^a^	4.49 ± 0.18 ^b^
Lys	5.73 ± 0.47 ^a^	5.07 ± 0.07 ^b^	3.97 ± 0.02 ^c^	4.76 ± 0.16 ^b^	4.09 ± 0.15 ^c^
Arg	10.24 ± 0.89 ^b^	10.08 ± 0.20 ^b^	10.10 ± 0.11 ^b^	11.67 ± 0.47 ^a^	10.02 ± 0.54 ^b^
Pro	3.33 ± 0.17 ^a^	3.30 ± 0.22 ^a^	3.23 ± 0.24 ^a^	3.21 ± 0.16 ^a^	3.06 ± 0.14 ^a^
Hydrophobic ^†^	33.64	32.96	33.76	34.12	33.23
Uncharged polar ^‡^	12.70	12.20	11.47	10.58	12.50
Basic ^⁋^	16.41	16.90	16.90	18.37	18.11
Acidic ^§^	27.55	27.36	27.94	26.74	26.42
Essential amino acids ^¶^	44.87	45.56	44.44	44.70	46.88
Aromatic amino acids ^ǁ^	12.16	10.51	11.75	13.33	11.54

^†^ Ala, Val, Ile, Phe, Pro, Gly and Met; ^‡^ Cys, Thr, Tyr; ^⁋^ Arg, His and Lys; ^§^ Asp and Glu; ^¶^ Thr, Val, Ile, Leu, Lys, His, Met, Phe, Tyr and Cys; ^ǁ^ Tyr and Phe. Data are expressed as the mean of three determinations ± standard deviation. In each column, values followed by different superscript letters are significantly different from each other (*p* < 0.05).

**Table 4:** Results of secondary structure content analysis using FT-IR and parameters of thermograms recorded by DSC of glutelin fraction.

Sample	Secondary Structures (%)				Thermostability				
	α-Helix	β-Sheet	β-Turn	Random Coil	*T*_o_ (°C)	*T*_e_ (°C)	*T*_p_ (°C)	*∆H* (J/g)	*T*_o_ (°C)
NR-glutelin	19.59	44.51	11.65	24.25	50.07	106.93	90.48	14.91	50.07
SR25-glutelin	21.15	42.57	12.35	23.92	45.57	127.86	92.04	14.88	45.57
SR50-glutelin	21.73	39.29	14.60	24.36	47.10	132.38	85.39	28.81	47.10
SR75-glutelin	33.91	39.64	9.64	24.45	51.80	141.79	88.26	29.19	51.80
SR100-glutelin	21.64	39.44	14.47	16.81	49.23	127.44	86.58	18.62	49.23

*T*_o_ and *T*_e_ are the initial and final temperatures of endothermic transition. *T*_p_ is the temperature at peak maximum and *∆H* is the enthalpy of denaturation.

## Abbreviations

FT-IR	Fourier transform infrared spectrometry
DSC	Differential scanning calorimetry
SeMetO	selenomethionine selenoxide
